# A systematic review of full economic evaluations of robotic-assisted surgery in thoracic and abdominopelvic procedures

**DOI:** 10.1007/s11701-023-01731-7

**Published:** 2023-10-16

**Authors:** Hamid Sadri, Michael Fung-Kee-Fung, Bobby Shayegan, Pierre Y. Garneau, Padina Pezeshki

**Affiliations:** 1 Department of Health Economic and Outcomes Research, Medtronic ULC, 99 Hereford St., Brampton, ON L6Y 0R3 Canada; 2 Champlain Regional Cancer Program Depts OB/GYN, Surgery, The Ottawa Hospital, 501 Smyth Rd, Ottawa, ON K1H 8L6 Canada; 3 Division of Urology, Department of Surgery, McMaster University, 50 Charlton Ave., Hamilton, ON L8N 4A6 Canada; 4 Surgical Department, Hôpital du Sacré-Coeur de Montréal, 5400 Boul Gouin O, Montréal, QC H4J 1C5 Canada; 5 Department of Clinical Research, Medtronic ULC, 99 Hereford St., Brampton, ON L6Y 0R3 Canada

**Keywords:** Systematic review, Robotic-assisted surgery, Cost-effectiveness analysis, Cost-utility analysis, Thoracic and abdominopelvic

## Abstract

This study aims to conduct a systematic review of full economic analyses of robotic-assisted surgery (RAS) in adults' thoracic and abdominopelvic indications. Authors used Medline, EMBASE, and PubMed to conduct a systematic review following the preferred reporting items for systematic reviews and meta‐analyses (PRISMA) 2020 guidelines. Fully published economic articles in English were included. Methodology and reporting quality were assessed using standardized tools. Majority of studies (28/33) were on oncology procedures. Radical prostatectomy was the most reported procedure (16/33). Twenty-eight studies used quality-adjusted life years, and five used complication rates as outcomes. Nine used primary and 24 studies used secondary data. All studies used modeling. In 81% of studies (27/33), RAS was cost-effective or potentially cost-effective compared to comparator procedures, including radical prostatectomy, nephrectomy, and cystectomy. Societal perspective, longer-term time-horizon, and larger volumes favored RAS. Cost-drivers were length of stay and equipment cost. From societal and payer perspectives, robotic-assisted surgery is a cost-effective strategy for thoracic and abdominopelvic procedures.

*Clinical trial registration* This study is a systematic review with no intervention, not a clinical trial.

**Supplementary Information:**

The online version contains supplementary material available at 10.1007/s11701-023-01731-7.

## Background

Modern surgery continues to save lives and open new possibilities in medicine [1]. The continual need and desire for improving clinical outcomes have led to technological and procedural advancements, including laparoscopy that revolutionised surgical procedures, enabling minimally invasive surgery (MIS) [2]. In this continuum, more than two decades ago, surgical robots were introduced and made available to the mainstream surgical field. Robotic-assisted surgery (RAS) has since transformed MIS [3, 4]. Historically, fields such as surgical oncology, urology, and gynaecology/oncology have led the way in developing RAS [5–9]. RAS has allowed improved optical visualisation and surgical manoeuvring for retraction, exposure, and tissue resection in these areas [10].

Today, RAS, particularly in urology (e.g., prostatectomy), is preferred and adopted by both patients and surgeons [11–13]. In the United States, 85% of prostatectomies [14, 15] and 50% of hysterectomies are performed robotically [16, 17]. RAS has been used in other surgical sites, including renal, bladder, colorectal, upper gastric, thoracic, cardiac, and head and neck surgery [18–22].

In recent years, a robotic surge of 15% in all procedures has been quoted [17]. The clinical benefits of RAS in various therapeutic fields have been documented in the literature [23, 24]. They include improved oncological outcomes, reduced mortality and perioperative complications, blood transfusion requirements, and length of hospital stay. Moreover, a shorter learning curve than laparoscopic techniques, consistency, enhanced instrument dexterity, precision, visualisation, and improved surgeon experience and comfort for the operators have been listed [25–27]. Nonetheless, the perceived upfront cost is a key barrier to adopting RAS [28].

Like any new advanced technology, RAS was more costly compared to conventional surgical procedures at inception. Indeed, several studies have solely examined the cost of RAS in various therapeutic areas [29–31].

Healthcare policymakers and payers are interested in a system-wide comparative full economic value assessment of a technology that considers both the cost and the outcomes of adopting a technology [32]. The question of the feasibility of technology is not how much it costs; rather, is it a good value for money?

In recent years, multiple cost analysis of RAS has been published while far less economic analysis has been conducted. The costing or cost consequence analysis emphasises the cost without consideration of the outcomes of a medical procedure or service. In contrast, a full economic analysis considers both cost and outcomes. There are four types of full economic evaluations for medical technologies and services. Cost-effectiveness analysis (CEA) and cost-utility analysis (CUA) consider both the cost and the clinical benefits (e.g., reduced blood pressure) or the health benefits (e.g., health-related quality of life) gains, respectively. Cost-minimisation analyses are a specific type of CEA where the outcomes of the comparator technologies are equal. Finally, in the cost–benefit analysis, the benefits of the technology or service are measured in monetary terms [33].

Cost-effectiveness and cost-utility studies have been published on RAS in several countries and from different perspectives. This study aims to conduct a systematic review of the full economic analysis (CEA and CUA) of RAS in thoracic and abdominopelvic surgeries.

## Methods

**Fig. 1:**
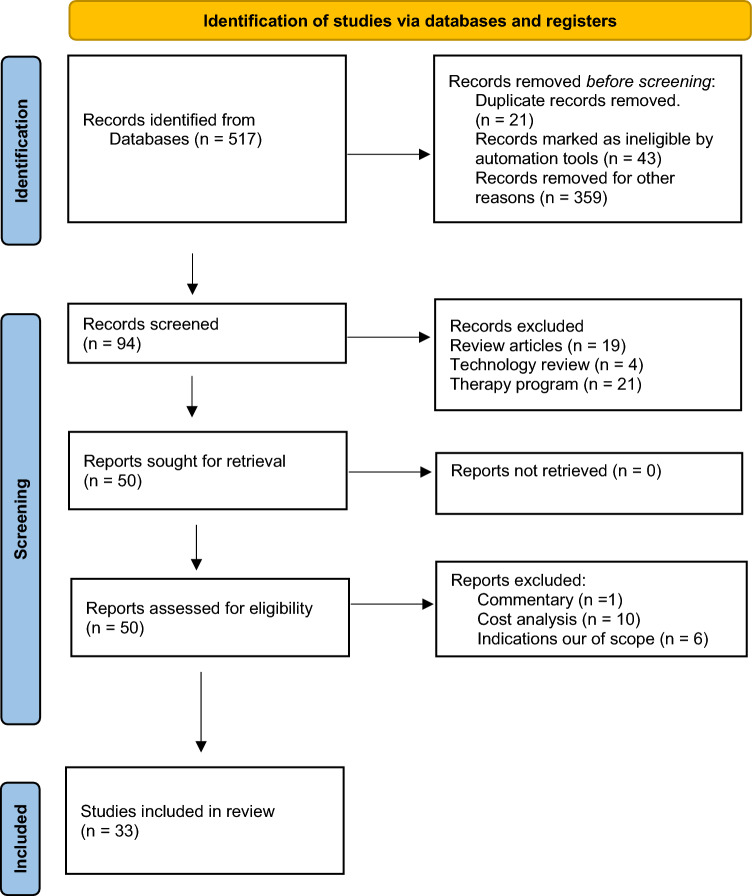
Preferred reporting items for systematic reviews and meta-analyses (PRISMA) flow chart

We conducted a systematic review (SR) using the preferred reporting items for systematic reviews and meta‐analyses (PRISMA) 2020 guidelines (Fig. 1). [34] We used the Embase, MEDLINE, and PubMed databases from 2000 to May 2023. Detailed search strategy and keywords are summarised in Supplemental Table S1. Two reviewers independently screened and assessed the title and abstract of all identified studies according to the following criteria:

### Inclusion criteria

o Scope: Robotic-assisted surgery in the thoracic, abdomen, and pelvic area, including urology, gynaecology, and others (defined as any other thoracic, abdominal, and pelvic procedure)
o Population: Adult patients with any diagnosis within scope who needed surgery
o Type of study: original full economic analysis: CEA and CUA, including studies alongside clinical trials
o Publications: full article published from the year 2000 in the English language

### Exclusion criteria

o Out of scope: Head, neck, and musculoskeletal procedures
o Study type: budget impact or financial impact analysis, cost analysis without outcome, clinical programs
o Publication type: abstracts, review articles, letters, commentary, not in the English language

Disagreements in the identification of related studies were deliberated to reach an agreement. A pre-specified EXCEL sheet (Microsoft Inc., Redmond, USA) was used for data extraction, including study year, country, type analysis, comparator, perspective, time horizon, the measure of effectiveness, cost and outcomes values, incremental cost, and effectiveness value, incremental cost-effectiveness ratio (ICER), discount rate, type of sensitivity analysis, main cost drivers, authors' comment.

## Reporting quality and risk of bias

The methodological quality and risk of bias were assessed using the risk bias of economic evaluations assessed using the Consensus on Health Economic Criteria (CHEC) extended checklist [35]. The CHEC contains guidelines for each criterion and scoring and can be used to evaluate all economic evaluations. The CHEC consists of 20 yes-or-no questions, one for each category. Per the assessment guidance, we scored 'yes' (one point) when we agreed that the study paid adequate consideration to a specific aspect. In the case of insufficient information in the publication, we scored 'no' (zero points). The total score was converted to a percentage and assigned a grade where ≤ 50%, 51–75%, 76–95%, and > 95% were considered low, moderate, good, and excellent quality, respectively.

Consistent with the recommendations and requirements for economic evaluation publications, we evaluated the included papers against the consolidated health economic evaluation reporting standards (CHEERS) 2022 criteria for reporting economic studies [36]. The checklist has 24 items in six domains: (1) title and abstract, (2) introduction, (3) methods, (4) results, (5) discussion, and (6) other (e.g., funding and conflict of interest). We answered each item with yes (adequately reported), partial yes (partly reported), and no (not reported/not applicable). The results were reported as a percentage of fulfilled criteria.

## Data synthesis

We followed the latest recommendations for cost-effectiveness systematic reviews published by the International Society for Pharmacoeconomics and Outcomes Research [37]. A meta-analysis was planned, but the implementation was deferred to the extracted available data homogeneity. General study characteristics, such as indication, comparator, type of analysis, outcomes, and perspectives, were summarised. Where pooling data was not possible, we reported the study by the surgical site as a descriptive narrative.

## Results

### Search Strategy and general characteristics

The initial search strategy identified 514 studies. After applying the inclusion and exclusion criteria, data from 33 selected studies were extracted. The general characteristics of the included studies are summarised in Table 1. There was heterogeneity regarding settings, conditions, and comparators. Six studies (18.2%) were from the USA [38–43]; five (15.2%) were from Spain [44–48]; three were from each Brazil [49–51] and Canada [52–54], followed by two studies from each of France [55, 56], UK [57, 58], Ireland [59, 60] and the Netherlands [61, 62]. Finland [63], Colombia [64], Germany [65], China [66], Thailand [67], Italy [68], Australia [69], and Sweden [70] comprised the remaining eight countries.

**Table 1:** Descriptive statistics of included studies

Parameter/		N	%	Reference(s)
Geography geography				
Country	USA	6	18.2	[38–43]
	Spain	5	15.2	[44–48]
	Brazil	3	9.1	[49–51]
	Canada	3	9.1	[52–54]
	France	2	6.1	[55, 56]
	Ireland	2	6.1	[59, 60]
	UK	2	6.1	[57, 58]
	Netherlands	2	6.1	[61, 62]
	Finland	1	3.0	[63]
	Columbia	1	3.0	[64]
	Germany	1	3.0	[65]
	China	1	3.0	[66]
	Thailand	1	3.0	[67]
	Italy	1	3.0	[68]
	Australia	1	3.0	[69]
	Sweden	1	3.0	[70]
Condition				
Cancer	Prostate	16	48.5	[41, 49, 50, 52, 55, 57–59, 62, 64, 67, 69, 70]
	Renal	4	12.1	[43, 51, 56, 65]
	Pancreas	3	9.1	[45, 47, 68]
	Rectal	2	6.1	[42, 48]
	Lung (NSCLC)	2	6.1	[39, 66]
	Bladder	2	6.1	[38, 61]
	Gastric	1	3.0	[45]
	Colon	1	3.0	[44]
Prolapse	Rectal	1	3.0	[63]
	Pelvic organ	1	3.0	[66]
Perspective				
	Healthcare system	12	36.4	[50, 52, 57–59, 64, 66, 67, 69]
	Payer*	8	24.2	[39–42, 46–49]
	Societal*	8	24.2	[39, 42, 44, 55, 56, 62, 68, 70]
	Hospital	7	21.2	[38, 43, 45, 51, 61, 63, 65]
Publication date				
Year	2023	1	3	[56]
	2022	7	21.2	[39, 46, 50, 55, 58, 62, 64]
	2021	7	21.2	[40, 42, 44, 49, 51, 66, 68]
	2020	5	18.2	[38, 45, 47, 48, 52]
	2019	2	6.1	[61, 63]
	2018	1	3.0	[43]
	2017	2	6.1	[53, 54]
	2016	1	3.0	[65]
	2015	1	3.0	[67]
	2014	1	3.0	[59]
	2013	2	6.1	[41, 57]
	2011	2	3.0	[70]
	2007	1	3.0	[69]

*NSCLC* none small cell lung cancer

*Some studies had both perspectives

All studies were conducted after 2007. Most studies (28/33) were conducted on various malignancy-related procedures, with radical prostatectomy as the most reported publications (16/33) [41, 49, 50, 52–55, 57–60, 62, 64, 67, 69, 70]. Other malignancies that required surgery were renal [43, 51, 56, 65], pancreas [46, 47, 68], rectal [42, 48], lung [39, 66], bladder [38, 61], gastric [45], colon [44]. Two studies reported rectal and pelvic organ prolapse [40, 63]. Twenty-seven studies used QALY to measure effectiveness [38, 39, 41, 42, 44–50, 52, 55, 57–59, 62, 63, 66, 66–70], and six studies used the clinical endpoint and complication rates [43, 51, 56, 61, 64, 65]. Nine studies used primary data from clinical trials [44–48, 63, 68, 70], and 24 used secondary data sources [38–43, 49–55, 57–62, 64–67, 69]. All studies used modelling suitable to the time horizon. Fourteen studies (42.4%) chose a longer time horizon between 5 and 20 years [49, 50, 52, 54, 55, 57–60, 62, 63, 66, 66, 67], and one study used a lifetime time horizon [41]. Six studies reported 1-year [39, 42, 46, 54, 69, 70], and six used a short-term (30–90-day) time horizon [38, 43, 51, 61, 64, 65]. Five studies did not report the time horizon [44, 45, 47, 48, 68]. Studies used different perspectives. Twelve studies were conducted from a healthcare system perspective [50, 52–54, 57–60, 64, 66, 67, 69]; eight studies used the payer perspective [39, 41, 42, 46–49, 66] while another seven used the societal perspective [39, 44, 55, 56, 62, 68, 70], of which two used both payer and societal perspectives [39, 42]. Seven studies used the hospital perspective (Table 2). [38, 43, 45, 51, 61, 63, 65].

**Table 2:** Summary of studies

Study	Country (currency)	Condition	Comparative procedures	Study design	Perspective	Time horizon	Outcomes measure	Incremental (QALY & %)	Incremental cost	Discount rate	Sensitivity analysis	Main determinant(s)	WTP threshold	ICER (cost /QALY)	Authors conclusion
Kukreja 2020 [38]	USA (2018 $)	Bladder cancer	RAS vs open cystectomy	Decision tree	Patient	90 days	QALY	0.32	$17,063	NA	one-way & two-way	complications, transfusion	NR	$2969	RAS is cost-effective
Michels 2019 [61]	Netherlands (2018 €)	Bladder cancer	RAS vs open radical cystectomy	Decision tree	Hospital	30–90 days	complications	30 days: − 0.08 90 days: − 0.04	€3,117 -€3,365	NA	one-way & 1000 sample probabilistic	OR time, LOS, equipment cost	NR	minor 30 days: €40,781 90 days: €83,528 major 30 days: €62,582 90 days: €37,007	RAS has fewer complications but is more costly
Ferri 2021 [44]	Spain (2018 €)	Colon cancer	RAS vs Lap right colectomy	Model based on an observational study	Societal	NR	QALY	0.105	€1,227.64	0–5%	5000 samples Monte Carlo simulations	NR	€20,000–€30,000	€11,691.81	RAS is cost-effective
Caruso 2020 [45]	Spain (2018 €)	Gastric cancer	RAS vs open gastrectomy	Model based on a prospective study	Hospital	NR	QALY	0.038	€4713.25	3%	5000 samples Monte Carlo simulations	NR	€20,000–€30,000	Dominant	RAS is cost-effective
Chen 2021 [91]	China (2019 $)	NSCLC	RAS vs open vs video-assisted thoracoscopy	Markov model	Healthcare system	5 years	QALY	0.28	RAS vs open $3,104.8 RAS vs video $4,006.8	3%	One-way & probabilistic	Cost, survival	$30,000	RAS vs open: $10,967.4 RAS vs video: $80,324.9	RAS is cost-effective vs open, but video-assisted is dominant
Heiden 2022 [39]	USA (2020 $)	NSCLC	RAS vs open vs video-assisted lobectomy	Decision tree	Payer & societal	1 year	QALY	0.00219	payer: $394.9 societal: $247.7	NA	One-way	Complication & conversion rates, case volume, LOS	$50,000–$150,000	Payer: $180,755.1 societal: $113,388.8	Thoracotomy is dominated, but RAS is not cost-effective
Caruso 2022 [46]	Spain (2018 €)	Pancreas cancer	RAS vs open enucleation	Decision tree based on clinical trial	Payer	1 year	QALY	0.0879	€2,617.8	NR	5000 samples Monte Carlo simulations	NR	€20,000–€30,000	€29,782.13	RAS is cost-effective
De Pastena 2021 [68]	Italy (2017 €)	Pancreas cancer	RAS vs open distal pancreatectomy	Model based on an observational study	Societal	NR	QALY	0.12	€6,534	NR	NR	NR	€4800	€4,221.50	RAS has a high probability to be cost-effective
Vicente 2020 [47]	Spain (2018 €)	Pancreas cancer	RAS vs Lap distal pancreatomy	model based on a prospective observational study	Payer	NR	QALY	0.062	€287.4	3%	Monte Carlo simulations	cost, LOS, utility	€20,000–€30,000	€4,636.65	RAS is cost-effective
Wang 2021 [40]	USA (2019 $)	Pelvic organs prolapse	RAS vs Lap sacrocolpopexy vs vaginal apical suspension	Markov model	Payer	5 & 10 years	QALY	1.12 & 4.9	$35,479	3%	One-way	Recurrence rate, cost, time horizon	$50,000	$35,470	RAS dominated at 5 years but is cost-effective at 10 years
Caicedo 2022 [64]	Columbia (2021 $)	Prostate cancer	RAS vs open radical prostatectomy	Decision tree	Healthcare system	90 days	complications	0.04	$2,035	NA	One-way	Cost and complication rates	$6370	$18,987	RAS is not cost-effective
Close 2013 [57]	UK (2010 £)	Prostate cancer	RAS vs Lap radical prostatectomy	discrete event simulation model	Healthcare system	10 years	QALY	0.08	£1,412	3.5%	One-way	Case volume, time-horizon	£30,000	£18,329	RAS is cost-effective at volumes > 150
Cooperberg 2013 [41]	USA (2009 $)	Prostate cancer	RAS vs Lap vs open vs 3D conformal radiation	Markov model	Payer	lifetime	QALY	no difference	various scenarios	3%	one-way & multi-way	recurrence to metastasis time	NR	NR	RAS is cheaper than radiation and other modalities
de Oliveira 2021 [49]	Brazil (2018 R$)	Prostate cancer	RAS vs open radical prostatectomy	Discrete event simulation model	Payer	5 years	QALY	0.41	R$11,759.2	5%	NR	NR	R$114,026.5	R$22,690.84	RAS is cost-effective
Farah 2022 [55]	France (2021 €)	Prostate cancer	RAS vs RAS + stereotactic body radiotherapy	Markov model	Societal	10 years	QALY	1.528	€507	4%	One-way & probabilistic	cost	€710	€332	RAS is cost-effective
Faria 2022 [50]	Brazil (2021 B$)	Prostate cancer	RAS vs open vs Lap radical prostatectomy	Markov model	Healthcare system	20 years	QALY	0.02 vs open 0.04 vs Lap	RAS vs open: R$-263 RAS vs Lap: R$-1,067	5%	Probabilistic	NR	× 1& × 3 GDP	RAS vs open R$4,518.74 RAS vs Lap R$3,13.34	RAS is cost-effective
Hohwu 2011 [70]	Sweden (2008 €)	Prostate cancer	RAS vs open radical prostatectomy	Economic evaluation based on a cohort study	Societal	1 year	QALY	− 0.0065	€4,506	NA	One-way	Cost, case volume	NR	Direct: €64,343 indirect: €13,514	RAS is not cost-effective in short-term
Labban 2022 [58]	UK (2019 £)	Prostate cancer	RAS vs open vs Lap radical prostatectomy	Markov model	Healthcare system	10 years	QALY	0.24 & 0.12	RAS vs open: £526 RA vs Lap: − £1,785	3.5%	One way & probabilistic	Case volume, LOS, OR time	£30,000	RAS vs open: dominant RAS vs Lap: £4,293	RAS is cost-effective
Lindenberg 2022 [62]	Netherlands (2019 €)	Prostate cancer	RAS vs Lap radical prostatectomy	Model	Societal	7 years	QALY	0.059	€2,029	cost 4% outcomes 1.5%	One-way & probabilistic	Cost, utility	€80,000	€34,206	RAS is cost-effective
O'Malley 2007 [69]	Australia (2007 $)	Prostate cancer	RAS vs open radical prostatectomy	Model	Healthcare system	1 year	QALY	0.1	$2,445.74	NA	One-way	NR	$42,000 & $76,000	$24,457.43	RAS is cost-effective
Parackal 2020 [52]	Canada (2019 $)	Prostate cancer	RAS vs open vs Lap radical prostatectomy	Markov model	Healthcare system	10 years	QALY	0.0662	$1,701	1.5%	One-way	utility, case volume	$50,0000 & $100,000	$25,704	RAS is a cost-effective
Ratchanon 2015 [67]	Thailand (2012 ฿)	Prostate cancer	RAS vs Lap radical prostatectomy	decision tree	Healthcare system	10 years	QALY	0.05	฿120,359	3%	One-way & 1000 sample Monte Carlo simulation	Case volume	฿160,000	฿2,407,180	RAS is not cost-effective for 100 cases/year but may be cost-effective at 385 cases
Teljeur 2014 [59]	Ireland (2015 €)	Prostate cancer	RAS vs open vs Lap radical prostatectomy	model	Healthcare system	10 years	QALY	NR	NR	NR	One-way	Urinary and sexual function, OR time	€26,700	€24,193	NR
Alberta Health 2017 [54]	Canada (2016 $)	Prostate cancer	RAS vs open radical prostatectomy	Markov model	Healthcare system	9 years	QALY	0.19	$8,541	5%	One-way & probabilistic	Utility, cost, biochemical recurrence	$50,000	$44,471	RAS is a cost-effective
Health Quality Ontario 2017 [53]	Canada (2016 $)	Prostate cancer	RAS vs open radical prostatectomy	Markov model	Healthcare system	1 year	QALY	0.001	$6,234	5%	One-way & probabilistic	Utility, cost, case volumes	50,000	$5.2 mil	RAS is not cost-effective
HIQA 2011 [60]	Ireland (2011 €)	Prostate cancer	RAS vs open vs Lap radical prostatectomy	Markov model	Healthcare system	5 years	QALY	0.09	€2,487	4%	One-way	Utility, cost	€20,000 to €40,000 scenarios	€26,647	Inconclusive
Quijano 2020 [48]	Spain (2018 €)	Rectal cancer	RAS vs Lap rectal resection	Model based on a prospective observational study	Payer	NR	QALY	0.195	€303.40	3%	Monte Carlo simulations,	Cost, LOS	€20,000—€30,000	€1,555.9	RAS is cost-effective
Simianu 2021 [42]	USA (2017 $)	Rectal cancer	RAS vs open vs Lap proctectomy	Decision tree	Payer & societal	1 year	QALY	0.00066	Societal: $497 payer: $983	NA	One-way & probabilistic	Cost, LOS	$100,000	Societal: $751,056 payer: $1,485,139	Open proctectomy dominated by Lap, RAS proctectomy may be cost-effective under certain conditions
Mäkelä-Kaikkonen 2019 [63]	Finland (2017 €)	Rectal prolapse	RAS vs Lap ventral mesh rectopexy	Prospective RCT	Hospital	2 to 5 years	QALY	2 years: 0.043 5 years: 0.104	€1,739.20	3%	One-way & multi-way	Case volume, technology lifetime	€43,500	2 years: €39,982 5 years: €16,707	RAS is cost-effective in long-term
Baghli 2023 [56]	France (2022 €)	Renal cancer	RAS vs open partial nephrectomy	Model based on retrospective analysis	Societal	1 year	Complications	0.11	€1,332	NA	One-way probabilistic	NR	NMB	€12,039	RAS is cost-effective
Buse 2016 [65]	Germany (2014 $)	Renal cancer	RAS vs open vs Lap partial nephrectomy	Decision tree	Hospital	Perioperative	Complications	RAS vs Lap: -0.47 RAS vs open: 0.13	$647	NA	5000 samples Monte Carlo simulation	Complication rates	Relative to the complications, treatment cost	RAS vs open: $5,005 Lap vs open: dominant	RAS is cost-effective
Buse 2018 [43]	USA (2018 $)	Renal cancer	RAS vs open partial nephrectomy	Decision tree	Hospital	30 days	Complications	0.12	NR	NA	2000 sample Monte Carlo	Case volume, renal score	NR	Dominant	RAS is a cost-effective
Garcia 2021 [51]	Brazil (2020 B$)	Renal cancer	RAS partial nephrectomy vs percutaneous cryoablation	Decision tree	Hospital	30 days	Complications	0.05	B$16,964	NA	One-way	Complication rate	NR	Dominated	PCA is Dominant and cost-saving in 30 days

*RAS* robotic-assisted surgery, *vs* versus, *Lap* laparoscopic, *QALY* quality-adjusted life years, *WTP* willingness to pay, *RCT* randomized controlled trial, *NA* not applicable, *NR* not reported, *LOS* length of stay, *OR* operating room

### Reporting quality and risk of bias assessment

The CHEC scores for methodological quality ranged from 45 to 95%, with the average score for the included studies being 79%. Five studies were classified as low-quality, where they focused on the clinical experience [47, 59, 64, 68, 69]. Seven studies were classified as moderate [40, 43, 45, 46, 51, 65, 70], and the remaining were graded as good or excellent. The details of the quality scores are presented in Supplementary Table S3.

The average report score for papers was 84.2% (71.4% to 92.9%). There was no demonstrable difference based on geographical region or publication date. The average score for each domain was 84.3% (71.4–91.9%). Primarily, the studies' lowest reporting scores were related to characterising heterogeneity (27.3%), approach and effect on patient and stakeholder engagement (12.1% and 51.5%, respectively), and characterising distribution effect (48.5%). These parameters may affect the accuracy of the analysis. The economic evaluations in the context of a health technology assessment had a higher score and adequately addressed stakeholder engagement and heterogeneity. (Supplemental Table S2).

### Therapeutic categorising

All studies determined the cost-effectiveness of RAS and the comparators within the respective jurisdiction's willingness-to-pay (WTP) acceptability thresholds. We categorised the studies in this systematic review into four primary speciality and subspecialty fields: urology, gastroenterology, gynaecology, and others (consisting of pancreas and lung cancer).

### Urology

#### Prostate cancer

Sixteen studies evaluated RAS's cost-effectiveness compared to alternate prostate cancer procedures. Twelve studies compared RAS to laparoscopic and/or open radical prostatectomy [41, 49, 50, 53, 54, 57–60, 62, 64, 67, 69, 70]. There was substantial heterogeneity among the studies, preventing the pooling of the data and conducting a meta-analysis or making a direct comparison. One study compared RAS prostatectomy with RAS plus stereotactic body radiation [55], and one had a comparator arm with 3D conformal radiation. [41] Overall, RAS provided superior outcomes compared to the comparator. RAS prostatectomy resulted in 0.05 to 1.5 QALY gain. All studies except one used the medium to long-term time horizons ranging from one year to a lifetime. One study used 90 90-day complication rate [64]. All studies used modelling to estimate the ICER using secondary data and concluded that RAS radical prostatectomy is a cost-effective strategy compared to conventional procedural care. Indeed, RAS was not deemed cost-effective only when short-term complication rates were the measure of effectiveness [64] and in a small single-centre cohort study [70].

#### Renal cancer

Three studies from the USA, Brazil, and Germany assessed the cost-effectiveness of RAS compared to open, laparoscopic nephrectomy and percutaneous cryoablation (PCA) for renal cancer [43, 51, 65]. All studies chose short-term complication rates as outcomes and calculated ICER by developing a simple decision tree. Two studies demonstrated that RAS is a cost-effective strategy compared to open surgery with an ICER of $5,005 from a German payer perspective and dominant from a US payer perspective [43, 65]. However, Garcia et al. reported that RAS was dominated vs. percutaneous cryoablation using a 30-day time horizon from a Brazil hospital payer perspective [51].

#### Bladder cancer

Two studies evaluated the cost-effectiveness of RAS compared to cystectomy in bladder cancer [38, 61]. Kukreja et al. reported an incremental cost of $2969 for a 0.3 QALY gain in 90 days and concluded that RAS is a cost-effective strategy [38]. In a study from the Netherlands, Michels et al. reported an 8% and 4% reduction in complications at 30 and 90 days [61]. They reported an ICER of €40,781 to €62,562 at 30 days and €83,528 to €37,007 at 90 days for minor and major complications, respectively. They concluded that RAS has a higher cost but lower complications and is a cost-effective strategy at 90 days. The analysis was sensitive to the length of hospital stay (LOS) and capital investment cost.

### Gastroenterology

#### Rectal cancer

One study from Spain compared RAS to laparoscopic rectal resection. The authors reported a 1.195 QALY gain for an incremental cost of €303, resulting in an ICER of €1555 below the WTP threshold, and concluded that RAS is cost-effective [48]. Simianu et al. compared RAS to open and laparoscopic proctectomy [42]. In their analysis, the laparoscopic procedure dominated the open procedure. RAS was cost-effective when the authors used real-world hospital resource use data from a societal perspective. RAS provided a 0.01 QALY gain compared to the laparoscopic procedure at a lower cost (-$470).

#### Rectal prolapse

A study from Finland examined RAS to laparoscopic ventral mesh rectopexy from a hospital perspective using short- and longer-term time horizons [63]. RAS provided better QALY gain of 0.043 at two years and 0.104 at five years, resulting in an ICER of €39,982 and €16,707, respectively, below the WTP threshold and more cost-effective in the long-term.

#### Colon cancer

In a study from Spain, Ferri et al. examined the feasibility of RAS compared to laparoscopic right colectomy from a societal perspective [44]. RAS provided 0.105 QALY gain at the incremental cost of €1227, resulting in an ICER of €11691 below the WTP threshold, making RAS a cost-effective strategy.

#### Gastric cancer

One study from Spain compared RAS to open gastrectomy, where RAS provided a 0.038 QALY gain, making RAS the dominant strategy [45].

### Gynaecology

#### Pelvic organs prolapse

Wang et al. conducted a cost-effectiveness analysis comparing RAS to sacrocolopropexy and vaginal apical suspension from a USA payer perspective [40]. RAS was dominated at five years but was cost-effective in 10 years with an ICER of $35,470.

### Others

#### Pancreas cancer

Two studies from Spain and one from Italy evaluated RAS's cost-effectiveness in pancreas cancer. Caruso et al. conducted CEA RAS compared to open enucleation. [46] An incremental cost of €2617 and 0.088 QALY gain resulted in ICER of €29,783. Similarly, De Pastena and Vicente reported a QALY gain of 0.12 and 0.062 at the incremental cost of €287 and €6534, resulting in an ICER of €4221 and €4636 in favour of RAS when compared to laparoscopic distal pancreatectomy. [47, 68] All studies concluded that RAS is a cost-effective strategy for pancreatic cancer.

#### Lung cancer

We included two studies in non-small cell lung cancer (NSCLC). Chen et al. compared RAS to open and video-assisted thoracoscopic from a healthcare system perspective over five years [66]. With a 0.28 QALY gain and ICER of $10,967, RAS was cost-effective compared to the open procedure, but the video-assisted procedure was dominant. Heiden et al. compared RAS to open and video-assisted thoracoscopic (VATS) lobectomy [39]. They reported small QALY gain and concluded that thoracotomy is dominated and RAS is not cost-effective from a payer perspective. However, with a WTP of $150,000, RAS was cost-effective from a societal perspective.

## Discussion

Our study provides a comprehensive systematic review of full economic analyses on robotic-assisted surgery. We identified 33 articles that evaluated the cost-effectiveness and cost-utility of RAS compared to open and laparoscopic surgical procedures across multiple disease sites. These studies were conducted worldwide and from various perspectives (Table 2).

Despite the diverse indications and jurisdictional perspectives, there were similarities among the studies. As the leading approach in economic analysis, all studies used economic modelling to estimate the cost-effectiveness of RAS. Despite variations, the main comparator in all indications was open surgery or laparoscopy. Five studies used one more alternative approach, including video-assisted surgery [39, 66], 3-D conformal radiation [41], radiotherapy [55], and percutaneous procedure [51]. The study's reporting was generally of high quality and reported the critical parameters of economic analysis. A few studies did not report on important parameters, including time-horizon [44, 45, 47, 48, 68], discount rate [45, 59, 68], which may impact the comparability of the results.

Overall, our results demonstrate that RAS has the propensity to be cost-effective in the described surgical procedures. Based on the authors' conclusions, 24 out of 33 (70%) studies determined that RAS is cost-effective or can be cost-effective under certain conditions. Only nine studies did not conclude that RAS was cost-effective or were inconclusive. Among these, four studies were in disciplines with comparatively limited experience with RAS [39, 42, 61, 71]. Factors influencing cost-effectiveness (conditional) included case volumes [57, 67], time horizon [40, 70], and societal perspective [42].

Our analysis revealed that RAS cost-effectiveness depends not just on the capital equipment cost but also on factors that improve the utility of robotic surgery over time. For instance, LOS and operating time (OT) are greatly influenced by the surgeon's experience, skill level, volumes, and postoperative pathways [61]. Michels et al. used a 30- and 90-day time horizon from a hospital perspective and concluded that RAS radical cystectomy for bladder cancer is not cost-effective unless the LOS is ≤ 4 days and OT is ≤ 175 min [61]. Nevertheless, they explained that with increased operator experience, the OT would decrease, and smaller OR teams might be needed to perform RAS procedures, potentially reducing procedure costs. Buse et al. examined open partial vs RAS nephrectomy. They reported fewer complications and nominally lower costs for RAS but only in centres where the surgeons had more experience with surgical robots, demonstrating the importance of the operator's RAS skills, which directly impact the feasibility of the technology [65].

Our findings are consistent with previous reviews of RAS. A review of the cost-utility analyses of RAS in radical prostatectomy showed that despite methodological limitations, over 80% of the studies found RAS to be cost-effective compared to open surgery [72]. Similarly, a review of RAS in multiple indications revealed that RAS is cost-effective compared to open surgery, but additional data for other comparators are needed [73].

To date, RAS has been primarily used in urology and gynaecology, including gynaecological malignancies. While numerous full economic studies have been published in urology, particularly prostatectomy, our literature search did not identify any cost-effectiveness study in gynecology, highlighting the need for such research. We could only identify one cost-effectiveness study for an enhanced recovery after surgery (ERAS) protocol [74]. However, multiple studies reported the superiority of RAS in gynaecology indications and potential cost savings due to higher efficiency [75, 76].

There are established guidelines for the economic evaluation of healthcare technologies in many jurisdictions. However, some studies deviated from such guidelines in their economic evaluations. For example, authors sometimes selected inadequate time horizons and focused on short-term results, typically alongside a clinical trial from a narrow perspective [39, 61, 64].

Studies have shown that there is an optimal timing for conducting an economic evaluation of medical technologies due to the learning curve for using advanced medical technologies [77]. Additionally, implementing new OR protocols and training requires change management [78]. Over time, increased user experience, improved surgical outcomes, reduced complication rates, and increased efficiencies (e.g., faster healing, shorter LOS) can be observed. For example, a report from Health Quality Ontario in 2017 doubted the value of RAS in prostatectomy [53]. The report's retrospective analysis of published literature found equal outcomes between RAS and conventional prostatectomy. In contracts, an Alberta health technology assessment in the same year included real-world evidence and concluded that RAS in prostatectomy provides good value for money with better outcomes, highlighting the importance of data sources in economic evaluations [54].

Furthermore, considering the real-world limitations and technical aspects is critical in assessing the feasibility and economic value of technologies like RAS. For instance, in the case of radical prostatectomy in Canada, non-academic and local centres often default to an open procedure with more complications and longer recovery times. Hence, the implementation, system realities, expertise, and capacity need to be considered for an informed health policy decision [79].

Analysis of the CEA/CUA studies of RAS draws attention to the fact that RAS improves outcomes and increases efficiency when implemented optimally, which is multifactorial. Comprehensive training and expertise of surgeons are fundamental to the success of an RAS program. Surgeons must undergo specialised training to acquire the necessary skills for operating robotic systems, including simulation-based training and proctorship programs [80]. A recent study demonstrated that 78.6% of general surgery program directors in the USA agreed that RAS should be integrated into residency training while balancing other necessary skills, ensuring that surgeons have the necessary RAS skills [81]. Furthermore, studies have shown the safety and feasibility of implementing ERAS protocols for RAS. One study showed that a same-day discharge program for RAS in radical prostatectomy is feasible, with no increase in rates of complications, unscheduled visits, or readmissions [82]. A cohesive team of experts is another critical success factor in an RAS program. A computational model based on historical data indicates that OR time and surgical team performance can be improved by systematically implementing surgical team composition. The study also highlights the importance of all surgical team members' individual and dependent performances [83].

Researchers have introduced equity as the fifth component of a quality healthcare system [84]. They have realised that providing high-quality patient care is not possible without addressing healthcare provider burnout and inequity. The COVID-19 pandemic exposed long-standing shortcomings and inequities in global healthcare systems, including Canada [85]. Despite efforts, the ageing population and rural–urban divide make equitable access to specialised and subspecialised care problematic. For example, the equity and quality of surgical procedures are inconsistent across regions, with most experienced surgeons often practising in large academic centres equipped with the latest technologies, which are not as accessible outside urban centres [86]. RAS could improve select procedures' efficiency, accuracy, and outcomes, democratising access to high-quality, specialised surgeries [87]. However, despite the broad use of RAS in various therapeutic areas (e.g., urology and gynaecology), nuances such as surgeon preference, surgical type, and technique exist. Studies have shown that RAS may be more economically advantageous for the surgeon compared to laparoscopy [88]. Furthermore, with the integration of artificial intelligence with medical technologies, specifically in RAS and the expansion of telesurgery, this impact may become more pronounced in the future [89, 90].

### Limitations

This study should be interpreted within the context of several limitations. Firstly, our search was confined to fully published economic evaluations written in English, thereby excluding potentially relevant conference abstracts in this field. Consequently, it is plausible that future abstracts may contribute additional insights into the subject matter. Moreover, our exclusion of certain articles in the musculoskeletal disease domain that focused on specialised surgical robots used for specific indications warrants acknowledgement. However, it is crucial to recognise the rapid growth of research in this area, with multiple specialised orthopaedic surgical robots expected to enter the market in the near future.

Furthermore, the body of literature pertaining to the economic evaluation of robotic-assisted surgery (RAS) remains limited. Given the escalating interest in RAS utilisation, an imperative need exists for comprehensive and accurate economic evaluations encompassing the full scope of its impact. Such evaluations would provide invaluable guidance to policymakers in making well-informed decisions regarding funding and reimbursement of RAS within their respective jurisdictions.

Importantly, this study primarily focuses on cost-effectiveness and cost-utility analyses. While it is exceedingly unlikely, it is plausible that there may have been cost minimisation or cost–benefit analyses that were not included in our search. Additionally, the articles included in this systematic review used differing key parameters, such as perspectives, comparators, data sources, and time horizons. Consequently, quantitative analysis and pooling data were impractical.

## Conclusion

From a social and payers' perspective, robotic-assisted surgery is a cost-effective strategy for thoracic and abdominopelvic procedures. Evidence suggests RAS improves the patient's quality of life compared to open surgical procedures.

### Supplementary Information

**Supplementary file 1: Table S1.** Search strategy and keywords.

**Supplementary file 2: Table S2.** PRISMA checklist.

**Supplementary file 3: Table S3.** The Consensus Health Economic Criteria (CHEC) List assessment.

**Supplementary file 4: Table S4.** Consolidated Health Economic Evaluation Reporting Standards 2022 (CHEERS 2022) Assessment.

## Data Availability

The data supporting this study's findings are available on request from the corresponding author.

## Abbreviations

RAS: Robotic-assisted surgery
CEA: Cost-effectiveness analysis
CUA: Cost-utility analysis
WTP: Willingness to pay
ICER: Incremental cost-effectiveness ratio
QALY: Quality-adjusted life years

## Declarations

### Permission to reproduce material from other sources

This study is a systematic review of published literature, and we did not use proprietary information from other sources.
