# Supplementary material for: A systematic review of full economic evaluations of robotic-assisted surgery in thoracic and abdominopelvic procedures
Source: J Robot Surg. 2023 Oct 16;17(6):2671–85. doi: 10.1007/s11701-023-01731-7 (PMC10678817; doi:10.1007/s11701-023-01731-7)
Supplement: Supplementary file 1 — Table S1. Search strategy and keywords. [file 11701_2023_1731_MOESM1_ESM.docx]

**Supplemental Table S1 - Search strategy and keywords**

|  | **Full-text journal articles from OVID system Embase and MEDLINE from the year 2000 to Mayc25, 2023** |  |
| --- | --- | --- |
| **#** | **Searches** | **Results** |
| 1 | (prostatectom* or nephrectom* or cystectom* or ((urinar* or ureter* or urethra* or urolog* or kidney* or prostate* or bladder* or genitourinar*) adj2 (remov* or dissect* or resect* or excision*))).ti,ab. | 289310 |
| 2 | (gynecolog* or gynaecolog* or hysterectom* or myomectom* or parametrectom* or ((surg* or remov* or dissection* or resection* or excision*) adj4 (uterus* or uterine* or parametrium*))).ti,ab. | 438926 |
| 3 | (((groin* or inguinal* or ventral or incisional or hiatal) adj hernia?) or (abdomin* or abdomen*) adj wall adj2 (hernia? or reconstruct* or repair*))).ti,ab. | 72611 |
| 4 | ((rouxeny or roux en y or roux eny or rouxen y) adj2 (bypass* or by-pass* or procedur* or surger* or surgic*)).ti,ab. | 27892 |
| 5 | (general adj surger*).ti,ab. | 35019 |
| 6 | (fundoplication* or (nissen adj2 operation*)).ti,ab. | 17928 |
| 7 | (gastrectom* or gastroresect* or hemigastrectom* or ((gastro gastric or stomach) adj2 (resect* or remov* or extirpation*))).ti,ab. | 88497 |
| 8 | ((sleeve* adj3 gastr*) or gastricsleeve* or sleevegastr*).ti,ab. | 26355 |
| 9 | (lapband* or ((lap* or gastric*) adj3 band*)).ti,ab. | 14823 |
| 10 | (cardioesophag* or cardio esophag* or (Heller* adj2 (myotom* or operation* or cardiomyotom*))).ti,ab. | 4777 |
| 11 | (cholecystectom* or ((gallbladder* or gall bladder*) adj2 (resect* or surger* or surgic* or remov*))).ti,ab. | 86183 |
| 12 | (splenectom* or (spleen* adj2 (resect* or surger* or surgic* or remov*))).ti,ab. | 61688 |
| 13 | (((((small or large) adj2 (intestine* or bowel*)) or colon*) adj2 (remov* or resect*)) or colectom*).ti,ab. | 67046 |
| 14 | (appendectom* or (appendix* adj2 (remov* or resect* or surger* or surgic*))).ti,ab. | 27743 |
| 15 | ((lysis adj2 adhesion*) or adhesiolys*).ti,ab. | 7657 |
| 16 | (((lower anterior* or abdominoperin*) adj2 (remov* or resect* or surger* or surgic)) or rectopex*).ti,ab. | 12598 |
| 17 | (hepatectom* or ((liver* or hepatic*) adj2 (remov* or resect*))).ti,ab. | 114421 |
| 18 | (pancreatectom* or pancreaticoduodenectom* or pancreatoduodenectom* or (Whipple* adj2 (procedur* or remov* or resect*)) or (pancrea* adj2 (remov* or resect*))).ti,ab. | 81582 |
| 19 | (thoracic adj surger*).ti,ab. | 44885 |
| 20 | (lobectom* or lymphadenectom* or esophagectom* or oesophagectom* or pneumonectom* or segmentectom* or thymectom* or (lung adj2 (resect* or surger* or surgic* or remov*))).ti,ab. | 214409 |
| 21 | (wedge adj2 (resect* or surger* or surgic* or remov*)).ti,ab. | 14835 |
| 22 | (mediastinal adj2 (resect* or surger* or surgic* or remov*)).ti,ab. | 3234 |
| 23 | robot*.ti,ab. | 165892 |
| 24 | (da Vinci* or daVinci*).ti,ab,kw,dm,dv,tn,mf,kf,hw,sh,fx,ec,xt. | 16144 |
| 25 | (Senhance* or Telelap* ALF* or ((ALFX* or ALF X*) adj5 (robot* or SOFAR* or TransEnterix* or Trans Enterix*))).ti,ab,kw,dm,dv,tn,mf,kf,hw,sh,fx,ec,xt,tx. or (ALFX* or ALF X*).dm,dv,tn,mf. | 289 |
| 26 | cost-effectiveness analysis/ or cost-utility analysis/ or exp economic evaluation/ | 424741 |
| 27 | ((cost adj1 (utility or effectiveness)) or economic* or quality adjusted life year* or QALY*).ti,ab. | 957052 |
| 28 | (UK or United Kingdom or Canada or Japan or Switzerland or Germany or Australia or New Zealand or Sweden or Netherlands or France or Denmark or Norway or South Korea or Italy or Finland or Spain or Belgium or Austria or Europe, Asia).ti,ab,cp,kw,kf,hw,in,sh. | 43730887 |
| 29 | (US or USA or United States).ti. | 455744 |
| 30 | 1 and (23 or 24 or 25) | 30521 |
| 31 | 2 and (23 or 24 or 25) | 9456 |
| 32 | (3 or 4 or 5 or 6 or 7 or 8 or 9 or 10 or 11 or 12 or 13 or 14 or 15 or 16 or 17 or 18 or 19 or 20 or 21 or 22) and (23 or 24 or 25) | 19840 |
| 33 | 30 and (26 or 27) and 28 | 446 |
| 34 | 31 and (26 or 27) and 28 | 232 |
| 35 | 32 and (26 or 27) and 28 | 450 |
| 36 | (30 or 31 or 32) and 29 | 296 |
| 37 | (pediatric* or paediatric* or child or children or infant or infants or toddler* or baby or babies or preschool* or preschool* or teenager* or teenager* or newborn* or newborn* or neonate* or neo nat* or young adult* or boy or boys or girl or girls or adolescen* or pre adolescen* or preadolescen*).ti,ab. | 6121281 |
| 38 | (conference* or congress* or meeting* or poster* or symposia* or symposium* or (oral* and (presentation* or session*)) or (scientific* and session*) or workshop* or workshop* or trade journal* or audio* or video*).dt,pt. or (abstract* or poster or posters or editorial* or letter or note or book or patent* or comment or comments or interview or interviews).dt,pt,ti. | 13872448 |
| 39 | ((animal or animals or avian* or beagle* or bovine* or bull or bulls* or cadaver* or calf or calve* or canine* or cat or cats or cattle or cow* or dog or dogs or equine* or feline* or ferret* or fish* or foal* or frog* or gerbil* or goat* or hamster* or horse* or invertebrate* or lamb* or mammal* or mare* or marmoset* or mice or minipig* or mini pig* or monkey* or mouse* or murine* or ovine* or pig or piglet* or pigs or porcine* or primate* or rabbit* or rodent* or sheep* or swine* or veterinar*) adj5 (experiment* or investigat* or methods or model* or randomi* or research or studies or study or subjects or trial or trials)).ti,ab,dt,pt,ct,kf,hw,sh. or (animal* or veterinar* or pet medicine*).so,jn,jx,jw. | 6845373 |
| 40 | (33 or 34 or 35 or 36) not (37 or 38 or 39) | 838 |
| 41 | limit 40 to yr=2000-current | 830 |
| 42 | english*.lg. or (article in press or "in data review" or in process).st. or aheadofprint.pp. | 73370558 |
| 43 | 41 and 42 | 783 |
| 44 | remove duplicates from 43 | **517** |
