# Supplementary material for: A systematic review of full economic evaluations of robotic-assisted surgery in thoracic and abdominopelvic procedures
Source: J Robot Surg. 2023 Oct 16;17(6):2671–85. doi: 10.1007/s11701-023-01731-7 (PMC10678817; doi:10.1007/s11701-023-01731-7)
Supplement: Supplementary file 3 — Table S3. The Consensus Health Economic Criteria (CHEC) List assessment. [file 11701_2023_1731_MOESM3_ESM.docx]

|  |  | **Kukreja 2020** | **Michels 2019** | **Ferri 2021** | **Baghli 2023** | **Caruso 2020** | **Chen 2021** | **Heiden 2022** | **Caruso 2022** | **De Pastena 2021** | **Vicente 2020** | **Wang 2021** | **Caicedo 2022** | **Close 2013** | **Cooperberg 2013** | **de Oliveira 2021** | **Farah 2022** | **Faria 2022** | **Hohwu 2011** | **Labban 2022** | **Lindenberg 2022** | **O'Malley 2007** | **Parackal 2020** | **Ratchanon 2015** | **Teljeur 2014** | **AHS 2017** | **HQO 2017** | **HIQA 2011** | **Quijano 2020** | **Simianu 2021** | **Mäkelä‑Kaikkonen 2019** | **Buse 2016** | **Buse 2018** | **Garcia 2021** |
| --- | --- | --- | --- | --- | --- | --- | --- | --- | --- | --- | --- | --- | --- | --- | --- | --- | --- | --- | --- | --- | --- | --- | --- | --- | --- | --- | --- | --- | --- | --- | --- | --- | --- | --- |
| **1** | **Is the study population clearly described?** | 🗸 | 🗸 | 🗸 | 🗸 | 🗸 | 🗸 | 🗸 | 🗸 | 🗸 | 🗸 | 🗴 | 🗸 | 🗸 | 🗸 | 🗸 | 🗸 | 🗴 | 🗸 | 🗸 | 🗸 | 🗸 | 🗸 | 🗸 | 🗸 | 🗸 | 🗸 | 🗸 | 🗸 | 🗸 | 🗸 | 🗸 | 🗸 | 🗸 |
| **2** | **Are competing alternatives clearly described?** | 🗸 | 🗸 | 🗸 | 🗸 | 🗸 | 🗸 | 🗸 | 🗸 | 🗸 | 🗸 | 🗸 | 🗸 | 🗸 | 🗸 | 🗸 | 🗸 | 🗸 | 🗸 | 🗸 | 🗸 | 🗸 | 🗸 | 🗸 | 🗸 | 🗸 | 🗸 | 🗸 | 🗸 | 🗸 | 🗸 | 🗸 | 🗸 | 🗸 |
| **3** | **Is a well-defined research question posed in answerable form?** | 🗸 | 🗴 | 🗴 | 🗴 | 🗴 | 🗴 | 🗴 | 🗴 | 🗴 | 🗸 | 🗸 | 🗴 | 🗸 | 🗸 | 🗸 | 🗴 | 🗸 | 🗸 | 🗸 | 🗸 | 🗸 | 🗸 | 🗸 | 🗴 | 🗸 | 🗸 | 🗸 | 🗸 | 🗸 | 🗴 | 🗴 | 🗴 | 🗴 |
| 4 | **Is the economic study design appropriate to the stated objective?** | 🗸 | 🗸 | 🗸 | 🗸 | 🗸 | 🗸 | 🗸 | 🗸 | 🗸 | 🗸 | 🗸 | 🗸 | 🗸 | 🗸 | 🗸 | 🗸 | 🗸 | 🗸 | 🗸 | 🗸 | 🗴 | 🗸 | 🗸 | 🗸 | 🗸 | 🗸 | 🗸 | 🗸 | 🗸 | 🗸 | 🗸 | 🗸 | 🗸 |
| **5** | **Are the structural assumptions and the validation methods of the model properly reported?** | 🗸 | 🗸 | 🗴 | 🗴 | 🗴 | 🗸 | 🗸 | 🗴 | 🗴 | 🗴 | 🗸 | 🗸 | 🗸 | 🗸 | 🗸 | 🗸 | 🗸 | 🗴 | 🗸 | 🗸 | 🗸 | 🗸 | 🗸 | 🗸 | 🗸 | 🗸 | 🗴 | 🗴 | 🗸 | 🗴 | 🗸 | 🗸 | 🗸 |
| 6 | **Is the chosen time horizon appropriate in order to include relevant costs and consequences?** | 🗴 | 🗸 | 🗸 | 🗸 | 🗴 | 🗸 | 🗸 | 🗸 | 🗴 | 🗴 | 🗸 | 🗴 | 🗸 | 🗸 | 🗸 | 🗸 | 🗸 | 🗸 | 🗴 | 🗸 | 🗴 | 🗸 | 🗴 | 🗴 | 🗸 | 🗸 | 🗸 | 🗴 | 🗸 | 🗴 | 🗴 | 🗴 | 🗸 |
| 7 | **Is the actual perspective chosen appropriate?** | 🗴 | 🗸 | 🗸 | 🗸 | 🗸 | 🗸 | 🗸 | 🗸 | 🗸 | 🗸 | 🗸 | 🗸 | 🗸 | 🗸 | 🗸 | 🗸 | 🗸 | 🗸 | 🗸 | 🗸 | 🗴 | 🗸 | 🗸 | 🗴 | 🗸 | 🗸 | 🗸 | 🗸 | 🗸 | 🗸 | 🗴 | 🗴 | 🗴 |
| 8 | **Are all important and relevant costs for each alternative identified?** | 🗸 | 🗸 | 🗸 | 🗸 | 🗸 | 🗸 | 🗸 | 🗴 | 🗴 | 🗴 | 🗸 | 🗴 | 🗸 | 🗸 | 🗸 | 🗸 | 🗸 | 🗸 | 🗸 | 🗸 | 🗸 | 🗸 | 🗸 | 🗸 | 🗸 | 🗸 | 🗸 | 🗸 | 🗸 | 🗸 | 🗸 | 🗸 | 🗸 |
| 9 | **Are all costs measured appropriately in physical units?** | 🗸 | 🗸 | 🗸 | 🗸 | 🗸 | 🗸 | 🗸 | 🗴 | 🗴 | 🗴 | 🗴 | 🗴 | 🗸 | 🗸 | 🗸 | 🗸 | 🗸 | 🗸 | 🗸 | 🗸 | 🗸 | 🗸 | 🗸 | 🗸 | 🗸 | 🗸 | 🗸 | 🗴 | 🗸 | 🗸 | 🗴 | 🗴 | 🗴 |
| 10 | **Are costs valued appropriately?** | 🗸 | 🗸 | 🗸 | 🗸 | 🗸 | 🗸 | 🗸 | 🗴 | 🗴 | 🗴 | 🗴 | 🗴 | 🗸 | 🗸 | 🗸 | 🗸 | 🗸 | 🗴 | 🗸 | 🗴 | 🗴 | 🗸 | 🗸 | 🗴 | 🗸 | 🗸 | 🗸 | 🗴 | 🗸 | 🗸 | 🗴 | 🗴 | 🗴 |
| 11 | **Are all important and relevant outcomes for each alternative identified?** | 🗸 | 🗸 | 🗸 | 🗸 | 🗸 | 🗸 | 🗸 | 🗸 | 🗸 | 🗸 | 🗸 | 🗸 | 🗸 | 🗸 | 🗸 | 🗸 | 🗸 | 🗸 | 🗸 | 🗸 | 🗸 | 🗸 | 🗴 | 🗸 | 🗸 | 🗸 | 🗴 | 🗸 | 🗸 | 🗸 | 🗸 | 🗸 | 🗸 |
| 12 | **Are all outcomes measured appropriately?** | 🗸 | 🗸 | 🗸 | 🗸 | 🗸 | 🗸 | 🗸 | 🗸 | 🗸 | 🗴 | 🗴 | 🗴 | 🗸 | 🗸 | 🗸 | 🗸 | 🗸 | 🗴 | 🗸 | 🗸 | 🗴 | 🗸 | 🗸 | 🗴 | 🗸 | 🗸 | 🗸 | 🗸 | 🗸 | 🗸 | 🗸 | 🗸 | 🗸 |
| 13 | **Are outcomes valued appropriately?** | 🗸 | 🗸 | 🗸 | 🗸 | 🗸 | 🗸 | 🗸 | 🗸 | 🗴 | 🗴 | 🗸 | 🗴 | 🗴 | 🗴 | 🗴 | 🗸 | 🗸 | 🗸 | 🗸 | 🗸 | 🗴 | 🗴 | 🗸 | 🗴 | 🗸 | 🗴 | 🗴 | 🗸 | 🗸 | 🗸 | 🗸 | 🗸 | 🗸 |
| 14 | **Is an appropriate incremental analysis of costs and outcomes of alternatives performed?** | 🗸 | 🗸 | 🗸 | 🗸 | 🗴 | 🗸 | 🗸 | 🗸 | 🗸 | 🗸 | 🗸 | 🗸 | 🗸 | 🗸 | 🗸 | 🗸 | 🗸 | 🗸 | 🗸 | 🗸 | 🗴 | 🗸 | 🗸 | 🗸 | 🗸 | 🗸 | 🗸 | 🗸 | 🗸 | 🗸 | 🗸 | 🗸 | 🗸 |
| 15 | **Are all future costs and outcomes discounted appropriately?** | 🗴 | 🗴 | 🗸 | 🗴 | 🗸 | 🗴 | 🗸 | 🗸 | 🗴 | 🗴 | 🗸 | 🗴 | 🗸 | 🗸 | 🗸 | 🗸 | 🗸 | 🗴 | 🗸 | 🗸 | 🗴 | 🗸 | 🗸 | 🗴 | 🗸 | 🗴 | 🗸 | 🗸 | 🗸 | 🗸 | 🗸 | 🗸 | 🗸 |
| 16 | **Are all important variables, whose values are uncertain, appropriately subjected to sensitivity analysis?** | 🗸 | 🗸 | 🗸 | 🗸 | 🗸 | 🗸 | 🗸 | 🗸 | 🗸 | 🗸 | 🗴 | 🗸 | 🗸 | 🗸 | 🗸 | 🗸 | 🗸 | 🗸 | 🗸 | 🗸 | 🗴 | 🗸 | 🗸 | 🗴 | 🗸 | 🗸 | 🗸 | 🗸 | 🗸 | 🗸 | 🗸 | 🗸 | 🗸 |
| 17 | **Do the conclusions follow from the data reported?** | 🗸 | 🗸 | 🗸 | 🗸 | 🗸 | 🗸 | 🗸 | 🗸 | 🗸 | 🗸 | 🗸 | 🗸 | 🗸 | 🗸 | 🗸 | 🗸 | 🗸 | 🗸 | 🗸 | 🗸 | 🗸 | 🗸 | 🗸 | 🗸 | 🗸 | 🗸 | 🗸 | 🗸 | 🗸 | 🗸 | 🗸 | 🗸 | 🗸 |
| 18 | **Does the study discuss the generalizability of the results to other settings and patient /client groups?** | 🗴 | 🗴 | 🗴 | 🗴 | 🗴 | 🗸 | 🗴 | 🗸 | 🗴 | 🗴 | 🗸 | 🗸 | 🗴 | 🗸 | 🗴 | 🗴 | 🗴 | 🗸 | 🗸 | 🗸 | 🗴 | 🗴 | 🗸 | 🗴 | 🗴 | 🗸 | 🗸 | 🗸 | 🗸 | 🗴 | 🗴 | 🗴 | 🗴 |
| 19 | **Does the article/report indicate that there is no potential conflict of interest of study researcher(s) / funder(s)?** | 🗸 | 🗸 | 🗸 | 🗸 | 🗸 | 🗸 | 🗴 | 🗸 | 🗸 | 🗸 | 🗸 | 🗸 | 🗸 | 🗴 | 🗸 | 🗸 | 🗴 | 🗸 | 🗸 | 🗸 | 🗸 | 🗸 | 🗸 | 🗴 | 🗸 | 🗸 | 🗸 | 🗸 | 🗸 | 🗸 | 🗸 | 🗸 | 🗸 |
| 20 | **Are ethical and distributional issues discussed appropriately?** | 🗸 | 🗸 | 🗸 | 🗸 | 🗸 | 🗸 | 🗸 | 🗸 | 🗸 | 🗸 | 🗸 | 🗸 | 🗸 | 🗸 | 🗸 | 🗸 | 🗸 | 🗸 | 🗸 | 🗸 | 🗸 | 🗸 | 🗸 | 🗸 | 🗸 | 🗸 | 🗸 | 🗸 | 🗸 | 🗸 | 🗸 | 🗸 | 🗸 |
|  | **Score (%)** | 80 | 85 | 85 | 80 | 70 | 85 | 85 | 75 | 55 | 55 | 70 | 55 | 85 | 85 | 85 | 95 | 90 | 75 | 95 | 95 | 45 | 90 | 85 | 45 | 95 | 90 | 95 | 80 | 95 | 80 | 70 | 70 | 75 |
|  | **Grade** | G | G | G | G | M | G | G | M | L | L | M | L | G | G | G | E | E | M | E | E | L | E | G | L | E | E | E | G | E | G | M | M | M |

**Table S3 – The Consensus Health Economic Criteria (CHEC) List assessment**

L: low, M: moderate, G: good, E: excellent, 🗸: yes, x: no
