# Supplementary material for: A systematic review of full economic evaluations of robotic-assisted surgery in thoracic and abdominopelvic procedures
Source: J Robot Surg. 2023 Oct 16;17(6):2671–85. doi: 10.1007/s11701-023-01731-7 (PMC10678817; doi:10.1007/s11701-023-01731-7)
Supplement: Supplementary file 4 — Table S4. Consolidated Health Economic Evaluation Reporting Standards 2022 (CHEERS 2022) Assessment. [file 11701_2023_1731_MOESM4_ESM.docx]

|  |  | **Kukreja 2020** | **Michels 2019** | **Ferri 2021** | **Baghli 2023** | **Caruso 2020** | **Chen 2021** | **Heiden 2022** | **Caruso 2022** | **De Pastena 2021** | **Vicente 2020** | **Wang 2021** | **Caicedo 2022** | **Close 2013** | **Cooperberg 2013** | **de Oliveira 2021** | **Farah 2022** | **Faria 2022** | **Hohwu 2011** | **Labban 2022** | **Lindenberg 2022** | **O'Malley 2007** | **Parackal 2020** | **Ratchanon 2015** | **Teljeur 2014** | **AHS 2017** | **HQO 2017** | **HIQA 2011** | **Quijano 2020** | **Simianu 2021** | **Mäkelä‑Kaikkonen 2019** | **Buse 2016** | **Buse 2018** | **Garcia 2021** | **Percentage** |
| --- | --- | --- | --- | --- | --- | --- | --- | --- | --- | --- | --- | --- | --- | --- | --- | --- | --- | --- | --- | --- | --- | --- | --- | --- | --- | --- | --- | --- | --- | --- | --- | --- | --- | --- | --- |
| **Title** | 1 | 🗴 | 🗸 | 🗸 | 🗸 | 🗸 | 🗸 | 🗴 | 🗸 | 🗸 | 🗸 | 🗴 | 🗴 | 🗸 | 🗴 | 🗸 | 🗸 | 🗴 | 🗸 | 🗴 | 🗴 | 🗴 | 🗸 | 🗸 | 🗴 | 🗴 | 🗴 | 🗴 | 🗸 | 🗸 | 🗸 | 🗴 | 🗸 | 🗸 | 57.6 |
| **Abstract** | 2 | 🗸 | 🗸 | 🗸 | 🗸 | 🗴 | 🗸 | 🗸 | 🗸 | 🗸 | 🗸 | 🗸 | 🗴 | 🗸 | 🗴 | 🗴 | 🗴 | 🗸 | 🗸 | 🗸 | 🗴 | 🗸 | 🗸 | 🗸 | 🗸 | 🗸 | 🗸 | 🗸 | 🗸 | 🗸 | 🗸 | 🗸 | 🗸 | 🗸 | 81.8 |
| **INTRODUCTION** |  |  |  |  |  |  |  |  |  |  |  |  |  |  |  |  |  |  |  |  |  |  |  |  |  |  |  |  |  |  |  |  |  |  |  |
| Background and objectives | 3 | 🗴 | 🗸 | 🗸 | 🗸 | 🗸 | 🗸 | 🗸 | 🗸 | 🗸 | 🗸 | 🗸 | 🗸 | 🗸 | 🗸 | 🗸 | 🗸 | 🗸 | 🗸 | 🗸 | 🗸 | 🗸 | 🗸 | 🗸 | 🗸 | 🗸 | 🗸 | 🗸 | 🗸 | 🗸 | 🗸 | 🗸 | 🗸 | 🗸 | 97 |
| **METHODS** |  |  |  |  |  |  |  |  |  |  |  |  | 🗸 |  |  |  |  |  |  |  |  |  |  |  |  |  |  |  |  |  |  |  |  |  |  |
| Health economic analysis plan | 4 | 🗸 | 🗸 | 🗸 | 🗸 | 🗸 | 🗸 | 🗸 | 🗸 | 🗸 | 🗸 | 🗸 | 🗸 | 🗸 | 🗸 | 🗸 | 🗸 | 🗸 | 🗸 | 🗸 | 🗸 | 🗸 | 🗸 | 🗸 | 🗸 | 🗸 | 🗸 | 🗸 | 🗸 | 🗸 | 🗸 | 🗸 | 🗸 | 🗸 | 100 |
| Study population | 5 | 🗸 | 🗸 | 🗸 | 🗸 | 🗸 | 🗸 | 🗸 | 🗸 | 🗸 | 🗸 | 🗸 | 🗸 | 🗸 | 🗸 | 🗸 | 🗸 | 🗸 | 🗸 | 🗸 | 🗸 | 🗸 | 🗸 | 🗸 | 🗸 | 🗸 | 🗸 | 🗸 | 🗸 | 🗸 | 🗸 | 🗸 | 🗸 | 🗸 | 100 |
| Setting and location | 6 | 🗸 | 🗸 | 🗸 | 🗸 | 🗸 | 🗸 | 🗸 | 🗸 | 🗸 | 🗸 | 🗸 | 🗸 | 🗸 | 🗸 | 🗸 | 🗸 | 🗸 | 🗸 | 🗸 | 🗸 | 🗸 | 🗸 | 🗸 | 🗸 | 🗸 | 🗸 | 🗸 | 🗸 | 🗸 | 🗸 | 🗸 | 🗸 | 🗸 | 100 |
| Comparators | 7 | 🗸 | 🗸 | 🗸 | 🗸 | 🗸 | 🗸 | 🗸 | 🗸 | 🗸 | 🗸 | 🗸 | 🗸 | 🗸 | 🗸 | 🗸 | 🗸 | 🗸 | 🗸 | 🗸 | 🗸 | 🗸 | 🗸 | 🗸 | 🗸 | 🗸 | 🗸 | 🗸 | 🗸 | 🗸 | 🗸 | 🗸 | 🗸 | 🗸 | 100 |
| Perspective | 8 | 🗸 | 🗸 | 🗸 | 🗸 | 🗸 | 🗸 | 🗸 | 🗸 | 🗸 | 🗸 | 🗸 | 🗸 | 🗸 | 🗸 | 🗸 | 🗸 | 🗸 | 🗸 | 🗸 | 🗸 | 🗸 | 🗸 | 🗸 | 🗸 | 🗸 | 🗸 | 🗸 | 🗸 | 🗸 | 🗸 | 🗸 | 🗸 | 🗸 | 100 |
| Time horizon | 9 | 🗸 | 🗸 | 🗴 | 🗸 | 🗸 | 🗸 | 🗸 | 🗸 | 🗸 | 🗴 | 🗸 | 🗸 | 🗸 | 🗸 | 🗸 | 🗸 | 🗸 | 🗸 | 🗸 | 🗸 | 🗸 | 🗸 | 🗸 | 🗸 | 🗸 | 🗸 | 🗸 | 🗸 | 🗸 | 🗸 | 🗸 | 🗸 | 🗸 | 93.9 |
| Discount rate | 10 | 🗴 | 🗴 | 🗸 | 🗴 | 🗸 | 🗸 | 🗴 | 🗴 | 🗴 | 🗸 | 🗸 | 🗴 | 🗸 | 🗸 | 🗸 | 🗸 | 🗸 | 🗴 | 🗸 | 🗸 | 🗴 | 🗸 | 🗸 | 🗴 | 🗸 | 🗸 | 🗸 | 🗸 | 🗸 | 🗸 | 🗴 | 🗴 | 🗴 | 57.6 |
| Selection of outcomes | 11 | 🗸 | 🗸 | 🗸 | 🗸 | 🗸 | 🗸 | 🗸 | 🗸 | 🗸 | 🗸 | 🗸 | 🗸 | 🗸 | 🗸 | 🗸 | 🗸 | 🗸 | 🗸 | 🗸 | 🗸 | 🗸 | 🗸 | 🗸 | 🗸 | 🗸 | 🗸 | 🗸 | 🗸 | 🗸 | 🗸 | 🗸 | 🗸 | 🗸 | 100 |
| Measurement of outcomes | 12 | 🗸 | 🗸 | 🗸 | 🗸 | 🗸 | 🗸 | 🗸 | 🗸 | 🗸 | 🗸 | 🗸 | 🗸 | 🗸 | 🗸 | 🗸 | 🗸 | 🗸 | 🗸 | 🗸 | 🗸 | 🗸 | 🗸 | 🗸 | 🗸 | 🗸 | 🗸 | 🗸 | 🗸 | 🗸 | 🗸 | 🗸 | 🗸 | 🗸 | 100 |
| Valuation of outcomes | 13 | 🗸 | 🗸 | 🗸 | 🗸 | 🗸 | 🗸 | 🗸 | 🗸 | 🗸 | 🗸 | 🗸 | 🗸 | 🗸 | 🗸 | 🗸 | 🗸 | 🗸 | 🗸 | 🗸 | 🗸 | 🗸 | 🗸 | 🗸 | 🗸 | 🗸 | 🗸 | 🗸 | 🗸 | 🗸 | 🗸 | 🗸 | 🗸 | 🗸 | 100 |
| Measurement & valuation of resources & costs | 14 | 🗸 | 🗸 | 🗸 | 🗸 | 🗸 | 🗸 | 🗸 | 🗸 | 🗸 | 🗸 | 🗸 | 🗸 | 🗸 | 🗸 | 🗸 | 🗸 | 🗸 | 🗸 | 🗸 | 🗸 | 🗸 | 🗸 | 🗸 | 🗸 | 🗸 | 🗸 | 🗸 | 🗸 | 🗸 | 🗸 | 🗸 | 🗸 | 🗸 | 100 |
| Currency, price date, and conversion | 15 | 🗸 | 🗸 | 🗸 | 🗸 | 🗸 | 🗸 | 🗸 | 🗸 | 🗸 | 🗸 | 🗸 | 🗸 | 🗸 | 🗸 | 🗸 | 🗸 | 🗸 | 🗸 | 🗸 | 🗸 | 🗸 | 🗸 | 🗸 | 🗸 | 🗸 | 🗸 | 🗸 | 🗸 | 🗸 | 🗸 | 🗸 | 🗸 | 🗸 | 100 |
| Rationale and description of model | 16 | 🗸 | 🗸 | 🗸 | 🗴 | 🗸 | 🗸 | 🗸 | 🗸 | 🗸 | 🗴 | 🗸 | 🗸 | 🗸 | 🗸 | 🗴 | 🗸 | 🗸 | 🗸 | 🗸 | 🗸 | 🗴 | 🗸 | 🗸 | 🗸 | 🗸 | 🗸 | 🗸 | 🗸 | 🗸 | 🗴 | 🗴 | 🗴 | 🗴 | 72.7 |
| Analytics and assumptions | 17 | 🗸 | 🗸 | 🗸 | 🗸 | 🗸 | 🗸 | 🗸 | 🗸 | 🗸 | 🗸 | 🗸 | 🗸 | 🗸 | 🗸 | 🗴 | 🗸 | 🗴 | 🗸 | 🗸 | 🗸 | 🗸 | 🗸 | 🗸 | 🗸 | 🗸 | 🗸 | 🗸 | 🗸 | 🗸 | 🗸 | 🗴 | 🗴 | 🗸 | 84.8 |
| Characterizing heterogeneity | 18 | 🗴 | 🗸 | 🗴 | 🗸 | 🗴 | 🗴 | 🗴 | 🗴 | 🗸 | 🗴 | 🗸 | 🗴 | 🗸 | 🗸 | 🗴 | 🗴 | 🗸 | 🗴 | 🗴 | 🗴 | 🗴 | 🗴 | 🗴 | 🗸 | 🗴 | 🗴 | 🗴 | 🗴 | 🗴 | 🗴 | 🗸 | 🗴 | 🗸 | 27.3 |
| Characterizing distributional effects | 19 | 🗴 | 🗸 | 🗴 | 🗸 | 🗸 | 🗸 | 🗴 | 🗴 | 🗸 | 🗴 | 🗸 | 🗴 | 🗸 | 🗴 | 🗴 | 🗸 | 🗴 | 🗸 | 🗸 | 🗸 | 🗴 | 🗴 | 🗴 | 🗸 | 🗸 | 🗸 | 🗸 | 🗴 | 🗸 | 🗴 | 🗴 | 🗴 | 🗸 | 48.5 |
| Characterizing uncertainty | 20 | 🗸 | 🗸 | 🗸 | 🗸 | 🗸 | 🗸 | 🗸 | 🗸 | 🗸 | 🗸 | 🗸 | 🗴 | 🗸 | 🗸 | 🗸 | 🗸 | 🗸 | 🗸 | 🗸 | 🗸 | 🗸 | 🗴 | 🗸 | 🗸 | 🗸 | 🗸 | 🗸 | 🗸 | 🗸 | 🗸 | 🗸 | 🗸 | 🗸 | 90.9 |
| Approach to engagement with patients and others affected by the study | 21 | 🗴 | 🗴 | 🗴 | 🗴 | 🗴 | 🗴 | 🗴 | 🗴 | 🗴 | 🗴 | 🗴 | 🗴 | 🗴 | 🗴 | 🗴 | 🗴 | 🗴 | 🗴 | 🗴 | 🗴 | 🗴 | 🗴 | 🗴 | 🗴 | 🗸 | 🗸 | 🗸 | 🗸 | 🗴 | 🗴 | 🗴 | 🗴 | 🗴 | 12.1 |
| **RESULTS** |  |  |  |  |  |  |  |  |  |  |  |  |  |  |  |  |  |  |  |  |  |  |  |  |  |  |  |  |  |  |  |  |  |  |  |
| Study parameters | 22 | 🗸 | 🗸 | 🗸 | 🗸 | 🗸 | 🗸 | 🗸 | 🗸 | 🗸 | 🗸 | 🗸 | 🗸 | 🗸 | 🗸 | 🗸 | 🗸 | 🗴 | 🗸 | 🗸 | 🗸 | 🗸 | 🗸 | 🗸 | 🗸 | 🗸 | 🗸 | 🗸 | 🗸 | 🗸 | 🗸 | 🗸 | 🗴 | 🗸 | 93.9 |
| Summary of main results | 23 | 🗸 | 🗸 | 🗸 | 🗸 | 🗸 | 🗸 | 🗸 | 🗸 | 🗸 | 🗸 | 🗸 | 🗸 | 🗸 | 🗸 | 🗸 | 🗸 | 🗸 | 🗸 | 🗸 | 🗸 | 🗸 | 🗸 | 🗸 | 🗸 | 🗸 | 🗸 | 🗸 | 🗸 | 🗸 | 🗸 | 🗸 | 🗸 | 🗸 | 100 |
| Effect of uncertainty | 24 | 🗸 | 🗸 | 🗸 | 🗸 | 🗸 | 🗸 | 🗸 | 🗸 | 🗸 | 🗸 | 🗸 | 🗸 | 🗸 | 🗸 | 🗴 | 🗸 | 🗸 | 🗸 | 🗴 | 🗸 | 🗸 | 🗸 | 🗸 | 🗸 | 🗸 | 🗸 | 🗸 | 🗸 | 🗸 | 🗸 | 🗸 | 🗸 | 🗸 | 93.9 |
| Effect of engagement with patients and others affected by the study | 25 | 🗸 | 🗸 | 🗸 | 🗸 | 🗸 | 🗸 | 🗸 | 🗸 | 🗸 | 🗸 | 🗴 | 🗴 | 🗴 | 🗴 | 🗴 | 🗴 | 🗴 | 🗴 | 🗴 | 🗴 | 🗸 | 🗴 | 🗴 | 🗸 | 🗸 | 🗸 | 🗸 | 🗸 | 🗸 | 🗴 | 🗴 | 🗴 | 🗴 | 51.5 |
| **DISCUSSION** |  |  |  |  |  |  |  |  |  |  |  |  |  |  |  |  |  |  |  |  |  |  |  |  |  |  |  |  |  |  |  |  |  |  |  |
| Study findings, limitations, generalizability, and current knowledge | 26 | 🗸 | 🗸 | 🗸 | 🗸 | 🗸 | 🗸 | 🗸 | 🗸 | 🗸 | 🗸 | 🗸 | 🗸 | 🗸 | 🗸 | 🗸 | 🗸 | 🗸 | 🗸 | 🗸 | 🗸 | 🗸 | 🗸 | 🗸 | 🗸 | 🗸 | 🗸 | 🗸 | 🗸 | 🗸 | 🗸 | 🗸 | 🗸 | 🗸 | 100 |
| **OTHER RELEVANT INFORMATION** |  |  |  |  |  |  |  |  |  |  |  |  |  |  |  |  |  |  |  |  |  |  |  |  |  |  |  |  |  |  |  |  |  |  |  |
| Source of funding | 27 | 🗸 | 🗸 | 🗸 | 🗸 | 🗸 | 🗸 | 🗸 | 🗸 | 🗸 | 🗸 | 🗸 | 🗸 | 🗸 | 🗸 | 🗸 | 🗸 | 🗸 | 🗸 | 🗸 | 🗸 | 🗸 | 🗸 | 🗸 | 🗸 | 🗸 | 🗸 | 🗸 | 🗸 | 🗴 | 🗸 | 🗸 | 🗸 | 🗸 | 97 |
| Conflicts of interest | 28 | 🗸 | 🗸 | 🗸 | 🗸 | 🗸 | 🗸 | 🗸 | 🗸 | 🗸 | 🗸 | 🗸 | 🗸 | 🗸 | 🗸 | 🗸 | 🗸 | 🗸 | 🗸 | 🗸 | 🗸 | 🗸 | 🗸 | 🗸 | 🗸 | 🗸 | 🗸 | 🗸 | 🗸 | 🗸 | 🗸 | 🗴 | 🗸 | 🗸 | 97 |
| **Percentage** |  | 78.6 | 92.9 | 85.7 | 89.3 | 89.3 | 92.9 | 82.1 | 85.7 | 75.0 | 82.1 | 89.3 | 71.4 | 92.9 | 82.1 | 71.4 | 85.7 | 78.6 | 85.7 | 82.1 | 85.7 | 78.6 | 82.1 | 85.7 | 89.3 | 92.9 | 92.9 | 92.9 | 92.9 | 85.7 | 82.1 | 71.4 | 71.4 | 85.7 |  |

**Table S4: Consolidated Health Economic Evaluation Reporting Standards 2022 (CHEERS 2022) Assessment**
